# Fluid dynamics between injections in incomplete anti-VEGF responders within neovascular age-related macular degeneration: a prospective observational study

**DOI:** 10.1186/s40942-022-00363-7

**Published:** 2022-03-08

**Authors:** Anthony Gigon, Antonio Iskandar, Chiara Maria Eandi, Irmela Mantel

**Affiliations:** Jules-Gonin Eye Hospital, Fondation Asile des Aveugles, Department of Ophthalmology, University of Lausanne, 15 Avenue de France, CP 5143, 1004 Lausanne, Switzerland

**Keywords:** Refractory neovascular age-related macular degeneration, Incomplete response, Anti-VEGF, Artificial intelligence, Optical coherence tomography, Subretinal fluid, Intraretinal fluid

## Abstract

**Background:**

The purpose of the study was to investigate the short-term response profile after an intravitreal injection (IVI) of anti-vascular endothelial growth factor (VEGF) in patients with neovascular age-related macular degeneration (nAMD) and incomplete response to anti-VEGF.

**Methods:**

In this monocentric prospective observational study, we recruited patients with incomplete response to anti-VEGF, defined as presence of subretinal fluid (SRF) and/or intraretinal fluid (IRF) on optical coherence tomography (OCT) for at least 6 months despite monthly anti-VEGF treatment. Each patient underwent complete ophthalmic exam and imaging study (including OCT, fluorescein angiography, indocyanine green angiography, OCT-angiography) the day of their scheduled monthly IVI. Intermediate visits were performed weekly thereafter (comprising ophthalmic exam and OCT), until week 4. Fluid metrics were quantified using an artificial intelligence-based algorithm at baseline and at each subsequent weekly visit. Main outcomes were residual fluid volumes of SRF and IRF for each time point, and its relative change after treatment. Particular interest was given to each patients’ nadir point, which was used for association analysis with imaging parameters.

**Results:**

A total of 28 eyes of 26 patients were included into the study. The maximal response was reached at 1.93 weeks on average. The relative fluid resolution at nadir point was 66 ± 36.7%, with quartile limits at 49.1%, 83%, and 96.1%, respectively. Mean residual fluid volume was 64.9 ± 128.8 µl at nadir point. Residual fluid was positively correlated with baseline SRF (r = 0.76, p < 0.0001) and larger pigment epithelium detachment (r = 0.65, p = 0.0001). Polypoidal choroidal vasculopathy was associated with larger residual fluid (p = 0.0013).

**Conclusions:**

Incomplete anti-VEGF responders in nAMD showed significant mean fluid resolution between injections, typically after 2 weeks. However, complete resolution was the exception, and the amount of residual fluid varied greatly. To understand the role of the unresponsive fluid, further studies are needed.

## Background

Age-related macular degeneration (AMD) is the leading cause of visual impairment in developed countries [1]. Among patients suffering of advanced AMD, its neovascular form (nAMD) represents around two thirds of cases [2]. The advent of intravitreal injections (IVI) of anti-VEGF agents marked a change of paradigm in the management of nAMD and its prognosis [3]. Anti-VEGF treatment is now well established as the first line treatment for nAMD [4, 5].

Despite good efficacy of anti-VEGF treatments in nAMD, some cases remain poor responders. Several terms have been used to qualify a suboptimal response to treatment, such as “incomplete anti-VEGF response”, “unresponsive”, “refractory to anti-VEGF”, or “resistant to anti-VEGF” to name a few [6, 7]. Although no consensus exists on the definition of this condition, “refractory nAMD” has been proposed to comprise cases with evidence of exudative activity after 6 or more monthly IVI [6, 8]. Signs of activity include subretinal fluid (SRF) or intraretinal fluid (IRF) on optical coherence tomography (OCT), or hemorrhages on fundus examination [6, 8]. Of note, the term refractory itself is unfortunate, as it may wrongly suggest a total absence of response to treatment, and not a partial or suboptimal effect. Therefore, in this study we used the term “incomplete responders”.

The proportion of eyes with incomplete response to anti-VEGF is not well defined and studies have shown a wide array of results depending on definitions, ranging from 5% up to 53% [9–11]. Exudative activity of nAMD, whether recurrent of persistent, has been linked to poorer visual outcome [12, 13]. Thus, there is some concern for these cases with fluid present despite maximal monthly retreatment with anti-VEGF.

A recent publication [14] has suggested that incomplete responders are in fact early responders with rapid recurrences. Indeed, little has been reported so far about the response behavior in between monthly IVI. This response profile might be indicative of the underlying pathogenic pathway of the residual fluid. In case of complete absorption and rapid recurrence, VEGF might be the main mediator. However, in case of non-resolution even under intensive anti-VEGF treatment the remaining fluid might be related to VEGF-independent pathogenic pathways [7].

The goal of the present study was to evaluate the short-term response profile and its inter-individual variability after anti-VEGF IVI in incomplete responders. In addition, we intended to investigate imaging factors predictive of the inter-IVI response profile.

## Methods

### Study protocol

This is a monocentric prospective observational study conducted in the tertiary referral hospital of Jules Gonin, Lausanne, Switzerland. The research methods and analysis plan adhered to the tenets of the Declaration of Helsinki and the research protocol was approved by the Ethical Committee (CER-VD 2017-02175).

Patients were prospectively screened during regular AMD clinics for incomplete response to anti-VEGF, defined by the presence of exudative signs on OCT for at least 6 months, namely IRF and/or SRF, despite monthly IVI. Patients had to have undergone anti-VEGF treatment for at least 12 months, following a Treat and Extend regimen or an Observe and Plan regimen [15]. In addition, monthly injections were required for the last 6 months regardless of the treatment regimen (due to exudative activity of the disease). Exclusion criteria comprised any confounding retinal disease other than nAMD which could be responsible for presence of fluid, signs of intraocular inflammation, and impossibility to acquire good image quality. Patients meeting inclusion criteria were instructed about the study and informed consent was obtained before inclusion into the study.

A baseline visit was performed, immediately prior to the next planned monthly IVI. The latter was administered on the same day, or exceptionally within the next 7 days. Follow-up visits were performed weekly after the IVI until week 4 (W1 to W4). At each visit, a complete ophthalmic exam was performed, comprising best corrected visual acuity (BCVA) using an Early Treatment Diabetic Retinopathy Study (ETDRS) chart, intraocular pressure, anterior segment exam, dilated posterior segment exam. Spectral-domain OCT (SD-OCT) and infrared (IR) images were obtained at each visit (Spectralis 6 × 6 mm macular cube, 49 scans), applying the inbuilt follow-up mode. In addition, color fundus photography, fundus autofluorescence (FAF) (Spectralis), fluorescein angiography (FA) (HRA Heidelberg), indocyanine green angiography ICGA (HRA Heidelberg), and OCT angiography (OCT-A) (Optovue, RTVue XR 100, 6 × 6 mm scans) were performed at baseline.

Fluid metrics were quantified using an artificial intelligence (AI)-based algorithm (RetinAI, Bern, Switzerland), whose performance has been demonstrated previously [16]. To this end, OCT volume data was exported as coded E2E files. They were imported into the AI algorithm which then performed the automated layer and fluid segmentations. This allowed for computing of IRF volumes, SRF volumes, and pigment epithelium detachment (PED) volumes, at each time point.

In addition, general patient information was retrieved, including age and gender, the number of previous IVI, treatment duration, the anti-VEGF drug currently used, and the number of previously performed drug switches. Furthermore, multimodal imaging analysis was performed in the following way: baseline OCT was analyzed for the presence or absence of: IRF, SRF, signs of polypoidal choroidal vasculopathy (PCV) (defined as an abrupt pigment epithelium detachment accompanied by double layer sign), reticular pseudodrusen, retinal pigment epithelium (RPE) atrophy (described as incomplete and complete RPE and outer retinal atrophy, according to recent consensuses [17, 18]), vitreomacular traction, and cells in the vitreous. Other measurements retrieved included central retinal thickness (CRT) (from Heidelberg Spectralis; automated CRT measurement) and subfoveal choroidal thickness (single measurement using Heidelberg Spectralis measurement tool on enhanced depth imaging scans). Information gathered from FA and ICGA included: the type of macular neovascularization (MNV type 1, type 2, type 3), signs of inflammation (hyperfluorescence of the disc, outer blood retinal barrier breakdown—late FA frames), presence of PCV (middle and late ICGA frames), the visibility of a choroidal feeder vessel (early ICGA frames), the presence of multifocal choroidal hyperpermeability (late ICGA frames). Furthermore, it was attempted to identify the type of drusen if present and visible, classifying as serous drusen, pachydrusen or basal laminar drusen, according to their appearance on color fundus photos, fundus autofluorescence and FA. If not visible, whether truly absent or masked, they were classified as absent. OCT-A scans were analyzed for the presence or absence of five parameters, which are known to be signs of MNV activity with this modality [19]: (1) a well-defined shape (lacy-wheel, sea-fan, or long filamentous linear vessel), (2) branching of the MNV (numerous branching into tiny capillaries or rare large mature vessels), (3) the presence of anastomoses or loops, (4) the aspect of the vessel termini (presence of peripheral arcades or dead tree appearance), and (5) the presence of a perilesional hyporeflective lesion on en-face imaging at the level of Bruch’s membrane.

### Outcomes

Outcome parameters included the values and their changes from baseline for BCVA, IRF, SRF, PED, for each time point. The nadir time point for minimal residual fluid volume of IRF plus SRF was determined, and associations with anatomical parameters were evaluated. Maximal response was analyzed at each eye’s individual time point where the response was highest (nadir time point). For eyes with no fluid reduction after the IVI, their relative response was considered to be 0% (no response). These values of the nadir time point were then used for the association analysis.

### Statistical analysis

Statistical analyses were performed in JMP software for Windows (version 8.0.1, SAS institute Inc, Cary, NC). Descriptive statistics were performed, and paired changes over time were analyzed using Wilcoxon signed-rank test (not normally distributed data). Association analyzes were performed using logistic regression, ANOVA test, and Pearson correlation analysis, according to categorical or continuous data.

P-values < 0.05 were considered statistically significant.

## Results

### Cohort demographics and baseline characteristics

A total of 28 eyes of 26 patients were included. Mean age was 80 ± 6.56 years (range 67 to 96 years). Gender distribution was 22 (79%) females and 6 (21%) males. The mean number of previous IVI was 54.0 ± 24.7 over a mean treatment period of 67.5 ± 31.0 months. Mean number of previous treatment switches was 1.0 (range 0 to 5). At baseline, the mean time from preceding IVI was 30.1 ± 7.7 days. The drug used at the time of the study was Ranibizumab in 11 eyes, Aflibercept in 16 eyes, and Bevacizumab in 1 eye.

At baseline of the study, the mean VA was 73.3 ± 12.8.

Disease activity at baseline comprised IRF in 15 eyes and SRF in 17 eyes. Multimodal imaging signs of the cohort are summarized in Table 1.

**Table 1:** Clinical signs on imaging

Imaging modality	Signs	# present (%)
OCT, CFP, IR, FAF	Serous drusen	2 (7.1)
	Reticular pseudodrusen	11 (39.3)
	Basal laminar drusen	9 (32.1)
	Pachydrusen	16 (57.1)
	Retinal fibrosis	5 (17.9)
	RORA	14 (50)
	Polyps (double hyperreflective layer within PED)	5 (17.9)
FA, ICGA	Polypoidal vasculopathy	2 (7.1)
	Multifocal choroidal hyperpermeability	1 (3.6)
	Feeder vessel	11 (39.3)
	MNV type (1/2/3)	25/2/1 (89.3/7.1/3.6)
OCT-A	Identifiable MNV	21 (75)
	Lacy-wheel shape MNV	3 (14.3)
	Seafan shape	6 (28.6)
	Long filamentous linear vessels	9 (42.9)
	No distinct pattern	3 (14.3)
	Numerous branching into small capillaries	4 (19)
	Anastomoses and loops	13 (61.9)
	Peripheral arcades	13 (61.9)
	Perilesional hyporeflective halo	11 (52.4)

*OCT* optical coherence tomography, *CFP* color fundus photograph, *IR* infrared image, *FAF* fundus autofluorescence, *RORA* retinal pigment epithelium and outer retinal atrophy, *PED* pigment epithelium detachment, *FA* fluorescein angiography, *ICGA* indocyanine green angiography, *MNV* macular neovascularization, *OCT-A* OCT-angiography

### Distributions of responses on weekly follow-up

**Fig. 1:**
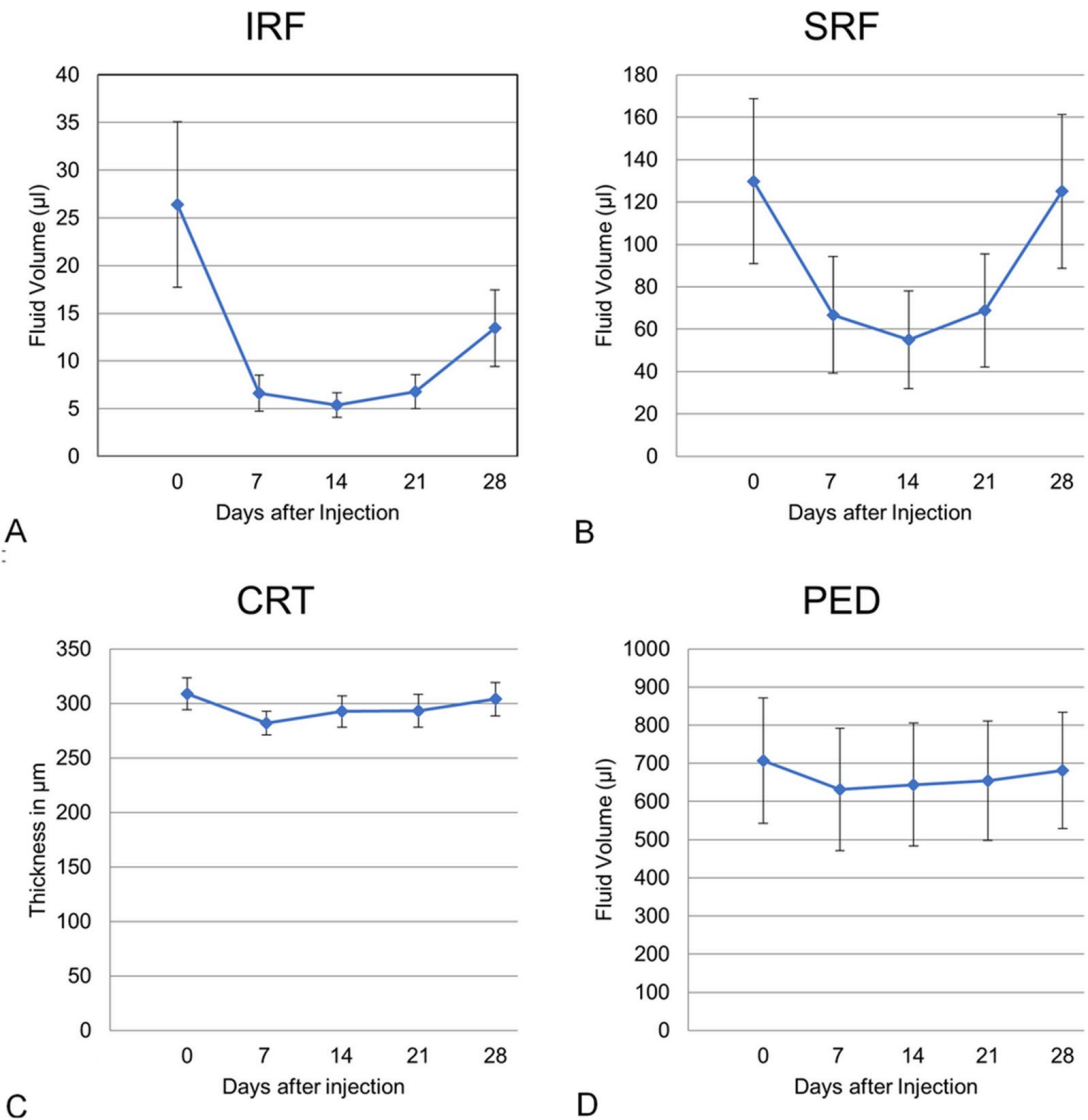
Weekly profile of fluid volumes after an intravitreal injection. Mean values with standard error bars. **A** Subretinal fluid (SRF). **B** Intraretinal fluid (IRF). **C** Pigment epithelium detachment (PED)

OCT metrics, specifically CRT, IRF, SRF, and PED volumes at baseline and at subsequent visits are summarized in Table 2 and graphically shown on Fig. 1. In summary, all these parameters displayed a statistically significant decrease at W1, W2, and W3 after the IVI. The statistically significant reduction was lost at W4.

**Table 2:** OCT parameters

OCT volumes	Baseline upfront anti-VEGF injection	Week 1	Week 2	Week 3	Week 4
CRT (µm)	309 ± 78	282 ± 57 (p < 0.01)	293 ± 76 (p = 0.02)	293 ± 80 (p = 0.01)	304 ± 81 (p = 0.37)
SRF (µl)	130 ± 506	67 ± 146 (p < 0.01)	55 ± 122 (p < 0.01)	69 ± 142 (p = 0.02)	125 ± 192 (p = 0.75)
IRF (µl)	26 ± 46	7 ± 10 (p = 0.02)	5 ± 7 (p = 0.02)	7 ± 10 (p = 0.03)	13 ± 21 (p = 0.07)
PED (µl)	707 ± 868	632 ± 849 (p < 0.01)	644 ± 853 (p < 0.01)	654 ± 826 (p < 0.01)	681 ± 808 (p = 0.08)

Values computed with the artificial intelligence-based algorithm at weekly visits (baseline, and week 1 to week 4)

*CRT* central retinal thickness, *PED* pigment epithelium detachment, *IRF* intraretinal fluid, *SRF* subretinal fluid

P-values computed with paired T-Test compared to baseline values

No statistically significant difference in VA was noticed throughout the follow-up period. Mean VA at baseline was 73.3 ± 12.8 letters. It was 73.4 ± 11.7 letters at W1 (p = 0.96), 73.7 ± 11.6 letters at W2 (p = 0.48), 74.0 ± 10.0 letters at W3 (p = 0.37), and 75.3 ± 9.7 letters at W4 (p = 0.72).

### Response profile for intra- and subretinal fluid

At baseline, mean algorithm-computed fluid was 156 ± 211.3 µl. The repartition per compartment was the following: 25 ± 45.5 µl of IRF and 129.4 ± 176.5 µl of SRF. Therefore, 83.8% of the total fluid at baseline was SRF.

**Fig. 2:**
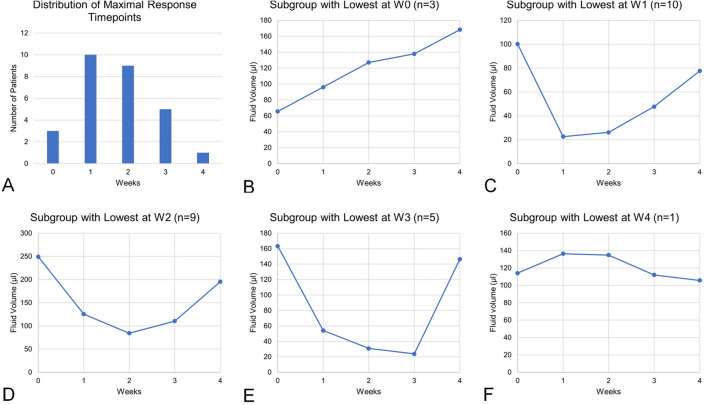
Time points at which maximum response is reached and subgroup profiles. **A** Distribution of patients per week of maximal response. **B**–**F** Profiles of response in terms of residual fluid volumes (IRF + SRF) as percentage of baseline, by subgroups

Referring to individual nadir time points, maximal fluid reduction was reached on average at 1.93 weeks. This nadir time point was reached in 10 (35.7%) eyes at W1, in 9 (32.1%) eyes at W2, in 5 (17.9%) eyes at W3, and in 1 (3.6%) eye at W4, while 3 (10.7%) eyes showed no improvement at all after the IVI (Fig. 2). The response profiles of each subgroup are displayed on Fig. 2.

**Fig. 3:**
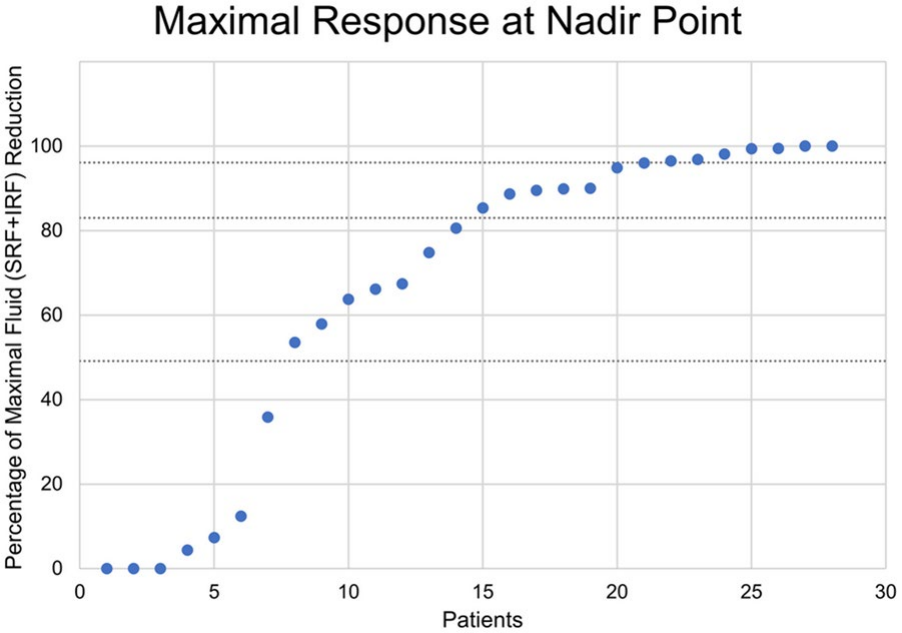
Distribution of intermediate fluid resolution. Measured at nadir in between anti-VEGF injections. 100% is equivalent to complete resolution, 0% is equivalent to absent improvement. Dotted horizontal lines represent quartile limits. *IRF* intraretinal fluid, *SRF* subretinal fluid

The nadir time point showed a mean IRF + SRF fluid reduction of 105 ± 143 µl (66 ± 36.7%), with quartile limits at 49.1%, 83%, and 96.1%, respectively. The distribution of relative response is shown on Fig. 3.

**Fig. 4:**
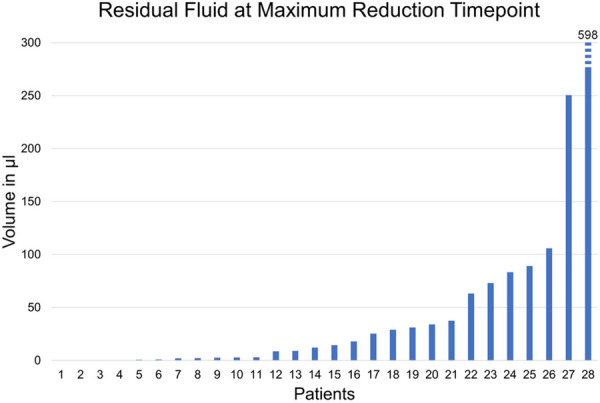
Amount of residual fluid. Total fluid (intraretinal fluid + subretinal fluid) measured at each patient’s nadir time point, in µl. Note that patient 28 is an outlier and his bar is cut from the graph

Residual fluid at the nadir time point was noted in 23 eyes (82.1%). Of the 17 eyes who presented SRF at baseline, 3 eyes showed a complete resolution of SRF at nadir, while 14 eyes (82.3%) still had remaining SRF. Of the 14 eyes with IRF at baseline, 3 eyes showed complete resolution at nadir, while 11 eyes (78.6%) displayed residual IRF. Mean algorithm-computed fluid volume of the eyes with residual fluid was 64.9 ± 128.8 µl. The repartition of residual fluid per compartment of these eyes was: 5.3 ± 6.7 µl IRF and 59.6 ± 128 µl SRF. In other words, 91.9% of residual fluid was SRF. The distribution of residual fluid is shown on Fig. 4.

### Association analysis

An association analysis for (a) maximal treatment response and (b) residual fluid amount was performed with respect to demographic, imaging or treatment factors. The results are summarized in Table 3.

**Table 3:** Association analysis

Continuous variables	Mean ± SD	Association with relative response	Association with residual fluid amount
Age	80 ± 6.74	r − 0.08, p = 0.69	r < 0.01, p = 0.99
Number of switches	1 ± 1.12	r 0.12, p = 0.53	r 0.06, p = 0.82
Number of IVI	53.3 ± 24.4	r 0.08, p = 0.69	r − 0.14, p = 0.46
Choroid thickness	173.4 ± 120.9	r − 0.01, p = 0.98	r 0.17, p = 0.37
CRT at baseline	308.9 ± 77.6	r 0.13, p = 0.50	r − 0.15, p = 0.44
IRF volume at baseline	26.4 ± 45.8	r − 0.29, p = 0.13	r − 0.06, p = 0.77
SRF volume at baseline	129.8 ± 506.2	r 0.02, p = 0.91	r 0.76, p < 0.0001
PED volume at baseline	697.9 ± 863.4	r 0.07, p = 0.70	r 0.65, p = 0.0001
Polyps on OCT	0.34 ± 0.9	r − 0.1, p = 0.62	r 0.04, p = 0.83
Total baseline fluid (µl)	155.6 ± 211.4	r − 0.04, p = 0.84	r 0.73, p < 0.0001

Categorical variables	# present (%)	Association with relative response	Association with residual fluid amount
Gender (female)	22 (79)	p = 0.056	p = 0.39
Medication (R/A/B)	11/16/1	p = 0.58	p = 0.46
Fibrosis	5 (17.9)	p = 0.56	p = 0.52
RORA on OCT	14 (50)	p = 0.07	p = 0.069
Reticular pseudodrusen	11 (39.2)	p = 0.37	p = 0.72
Feeder vessel on ICGA	11 (39.2)	p = 0.55	p = 0.44
Pachydrusen	15 (53.6)	p = 0.76	p = 0.74
Basal laminar drusen	8 (28.9)	p = 0.15	p = 0.36
Presence of IRF at baseline	15 (53.6)	p = 0.14	p = 0.075
Presence of SRF at baseline	18 (64.3)	p = 0.47	p = 0.09
PCV on ICGA	2 (7.1)	p = 0.95	p = 0.001
Well-defined shape on OCT-A	18 (85.7)	p = 0.7	p = 0.92
Presence of numerous branching on OCT-A	4 (19)	p = 0.22	p = 0.59
Presence of anastomoses/loops on OCT-A	13 (61.9)	p = 0.36	p = 0.18
Presence of peripheral arcades on OCT-A	13 (61.9)	p = 0.94	p = 0.21
Presence of perilesional hyporeflective halo on OCT-A	11 (52.4)	p = 0.54	p = 0.5

Association analysis between variables and relative response/residual fluid in terms of the sum of intra- and subretinal fluid volume at its strongest response time point. Pearson correlation test was used for continuous variables and ANOVA for categorical variables

*IVI* intravitreal injection, *CRT* central retinal thickness, *IRF* intraretinal fluid, *SRF* subretinal fluid, *PED* pigment epithelium detachment, *OCT* optical coherence tomography, *R* ranibizumab, *A* aflibercept, *B* bevacizumab, *RORA* retinal pigment epithelium and outer retinal atrophy, *ICGA* indocyanine green angiography, *PCV* polypoidal choroidal vasculopathy, *OCT-A* OCT-angiography

Patients’ demographics such as age and sex were not linked with response or residual fluid patterns, although there was a trend for more fluid resolution in male patients (p = 0.056). Furthermore, no association was found between preceding treatment characteristics, including time from last injection, drug type, time from treatment initiation, and number of past drug switches.

Similarly, no statistically significant associations with imaging parameters were found to explain relative treatment response.

However, residual fluid (sum of IRF and SRF), was positively correlated with baseline SRF (r = 0.76, p < 0.0001) and larger PED (r = 0.65, p = 0.0001). In other words, the higher SRF or PED volumes were at baseline, the more residual fluid was present.

Of note, some imaging parameters were present in insufficient numbers and therefore excluded from the association analysis. These included presence of serous drusen, multifocal choroidal hyperpermeability, and MNV type. Presence of PCV on ICGA also fell into this category (present in two eyes on ICGA), but given its well-known origin of potential refractory fluid, it was kept into the analysis. Indeed, there was one outlier with 598 µl residual fluid at week 2 (nadir) which also presented a PCV lesion on ICGA, which induced a statistically significant association (p = 0.0013) between PCV and residual fluid despite the low numbers.

## Discussion

This study showed the response profiles of incomplete anti-VEGF responders in nAMD, in terms of OCT metrics between monthly anti-VEGF IVI. An AI-based algorithm computed IRF, SRF, PED volumes and showed significant fluid reduction between injections, with its maximum typically 2 weeks after injection, and relapse at week 4. However, despite statistically significant resolution, most eyes still showed some residual fluid (IRF + SRF) at the nadir time point, and a proportion of eyes showed only little or no fluid reduction at all. Thus, these patients with incomplete response appeared to be a heterogeneous group varying between non-responding residual fluid and short-term responders.

To our knowledge, this is the first study specifically designed to evaluate short-term response on nAMD patients with incomplete response to anti-VEGF, using an automated quantitative approach to measure pathological fluids. Short-term response to IVI treatment in nAMD has previously been studied in terms of CRT [14]. Bontzos et al. prospectively evaluated the short-term effect of IVI in nAMD in general, with weekly SD-OCT after ranibizumab injection [14]. Their cohort consisted of 48 eyes, both treatment naïve and previously treated patients. They assessed the response to IVI treatment in terms of CRT and qualitative analysis of presence of fluid, with a semi-quantitative scale ranging from 1 to 4, according to the amount of fluid as appreciated by a human reader. Although their study population consisted of all nAMD cases and not only incomplete responders, their observation of a CRT nadir point 2 weeks after the IVI for non-naïve patients corresponds with our findings. However, they report little about the residual fluid which is unresponsive, or about the number of cases which could be considered incomplete responders.

In our cohort incomplete anti-VEGF responders only were included. The cohort included a surprisingly high proportion (89%) of type 1 MNV. Type 1 MNV has been reported to be the most frequent type of MNV, [20] but our findings seem especially high. This may suggest an increased proportion in type 1 MNV in refractory patients. However, this study was not designed to evaluate this association and the lack of a control group prevents from drawing definitive conclusions in that regard.

During IVI treatment with anti-VEGF, a mechanism of drug tolerance may induce suboptimal response to treatment [21]. Switching anti-VEGF therapy is thus a well-recognized strategy to encounter for this [22, 23]. Most patients in our cohort (85.7%) had had a medication switch before the inclusion into the study. Thus, it seems unlikely—although not completely excluded—that this mechanism would be relevant in this study.

When considering incomplete anti-VEGF response in nAMD, an anatomically special situation (such as PCV) or a masquerading differential diagnosis can be the cause of the suboptimal response. Indeed, PCV has been shown to be potentially resistant to anti-VEGF alone [24] and a combination treatment of anti-VEGF and photodynamic therapy (PDT) may be proposed [24]. In our cohort only two patients presented signs of PCV. This number is low, and possibly influenced by our routine use of ICGA in addition of FA, before initiating anti-VEGF treatment. Thus, PCV is frequently recognized early. After adjuvant PDT, these patients were no longer eligible for our study, thus not reaching the screening pool for incomplete response.

Some masquerading pathologies could be the origin of unresponsive fluid on OCT, such as central serous chorioretinopathy (CSC), epiretinal membranes, vitreomacular traction syndrome, or inflammatory causes [7, 25]. Therefore, we carefully analyzed the baseline multimodal imaging in order to detect any signs related to these pathologies. There were no relevant findings. However, we noticed a high prevalence of pachydrusen (16 eyes of 28). Although the choroid was not particularly thick (mean 173.4 ± 120.9 µm), it may indicate some diagnostic overlap with pachychoroid disorders. Nevertheless, no associations were found with residual fluid, or maximal response.

The main interest of the study focused on the fluid profile in between injections. The results showed significant fluid reduction in all compartments, with its mean maximum after approximately 2 weeks. However, it is interesting to note that complete resolution was infrequent (17.9%), and some eyes (10.7%) did not show any improvement at all. In other words, there was a large variation of the relative response profile, and even in between injections, residual fluid was the most frequent scenario, suggesting a role of other pathogenic pathways than VEGF.

However, it is unclear what quantity of residual fluid is clinically relevant. Indeed, the question of fluid tolerance remains open, with no definitive consensus on whether and how much persistent fluid could be accepted without functional consequences. The FLUID study has shown no difference in VA at 24 months if patients with SRF < 200 µm were left untreated. However, the difference in mean number of injections in comparison with the intensive treatment arm was so small that it is difficult to draw conclusions [26]. Other studies have also demonstrated that unresponsive SRF may still allow for good visual outcomes [27, 28]. It has been hypothesized that SRF could be a sign of a viable choriocapillaris and neovascular network, providing nutrients to the overlying RPE and preventing atrophy and loss of function [29]. Despite this, we are unaware of any studies evaluating the outcome over more than 3 years.

There is a general agreement that care should be taken to avoid undertreatment, as it plays a major role in the prognosis of nAMD [30]. Some advocate maximal treatment of any residual fluid [31]. Interestingly, a recent AI-based quantitative analysis of subretinal fluid from the FLUID study showed a negative correlation between SRF and BCVA [32]. The authors relativize the findings of the FLUID study and highlight how more precise fluid measurements can lead to different conclusions.

In contrast, the presence of any residual IRF is generally accepted as indicator for treatment need, as its presence is associated with poorer visual outcomes compared to SRF [27, 33–35].

Residual fluid despite maximal anti-VEGF treatment may indicate a VEGF independent source of fluid coexisting with the VEGF pathway in patients with incomplete anti-VEGF response. Therefore, it might be important to recognize its quantity and type, measured at the correct time point between injections, in order to potentially orientate the patient towards adjuvant treatment, if available. In this sense it is interesting to mention previous studies about the development of artificial intelligence-based algorithms to predict the response to anti-VEGF based on OCT scans in nAMD [36, 37].

AMD is a complex disease and its pathophysiology combines many different pathways, including among others, inflammatory molecules, the complement system, and lipoprotein metabolism, in addition to the influence of genetic susceptibility and environmental factors [38]. On that matter, we have previously reported an increase in inflammatory and vasoproliferative biomarkers in the aqueous humor of patients with incomplete response to anti-VEGF (some of them being included in this study as well), compared to a control group of standard nAMD patients [7] supporting the idea that other (anti-VEGF independent) mechanisms may be implicated. These findings may justify future studies about adjuvant anti-inflammatory treatment in this selective set of patients.

Demographic and treatment factors did not show any association with the relative response of residual fluids, which was somewhat surprising as we expected to find some biomarkers. However, residual fluid amounts were associated with baseline SRF amount, PED volume, and the presence of PCV on angiography. SRF is the dominating element in the sum of SRF and IRF. Indeed, in terms of volume, SRF represented 83.1% of total fluid (IRF + SRF) volume at baseline, and 91.9% of residual fluid volume. It is therefore not surprising that larger amounts of baseline SRF were associated with larger residual fluid volume.

Larger PED volumes at baseline were also associated with more residual fluid. This finding is in accordance with the literature, as the presence of PED has often been linked with more frequent injections need, and with poorer prognosis [29]. However, it is unclear whether the residual fluid is related to the structural form of the PED, leading to mechanical elevation of the photoreceptors, or related to higher exudative activity.

As mentioned above, due to the selection process of the cohort, only two eyes presented PCV on ICGA. However, its presence was associated with more residual fluid, particularly due to one outlier case. Although the association was statistically significant, conclusions must be handled with care due to the small number.

We acknowledge that 28 eyes with nAMD and incomplete response to anti-VEGF is a limited number of participants. However, the prospective design of the study, selecting specifically patients with incomplete response to anti-VEGF, and the detailed imaging documentation are some strengths.

In addition, the use of an AI-based algorithm to objectively quantify and categorize fluid compartments, allowed for detailed evaluation of the OCT imaging data with high precision.

## Conclusions

In conclusion, the study showed that a majority of nAMD patients with incomplete response to anti-VEGF displayed some short-term response to treatment, with a maximum response after approximately 2 weeks and an early relapse at week 4. However, this response was frequently incomplete throughout the weekly analyzed study period, with permanent presence of residual fluid to a variable degree in most patients. A small proportion of patients showed little or no fluid reduction in between injections, with predominantly unresponsive fluid. This highlights the heterogeneity of the group of nAMD patients with incomplete response to anti-VEGF, which suggests that many different factors come into play, including presumably anti-VEGF independent mechanisms.

## Data Availability

The datasets used and/or analyzed during the current study are available from the corresponding author on reasonable request.

## Abbreviations

AMD: Age-related macular degeneration
nAMD: Neovascular AMD
IVI: Intravitreal injections
VEGF: Vascular endothelial growth factor
SRF: Subretinal fluid
IRF: Intraretinal fluid
OCT: Optical coherence tomography
W1–W4: Week 1 to week 4
BCVA: Best corrected visual acuity
ETDRS: Early treatment dibabetic retinopathy study
SD-OCT: Spectral-domain OCT
IR: Infrared
FAF: Fundus autofluorescence
ICGA: Indocyanine green angiography
OCT-A: OCT angiography
AI: Artificial intelligence
PED: Pigment epithelium detachment
PCV: Polypoidal choroidal vasculopathy
RPE: Retinal pigment epithelium
CRT: Central retinal thickness
MNV: Macular neovascularization
CSC: Central serous chorioretinopathy
